# Assessment of Subject and Physician Satisfaction after Long-Term Treatment of Glabellar Lines with AbobotulinumtoxinA (Dysport^®^/Azzalure^®^): Primary Results of the APPEAL Noninterventional Study

**DOI:** 10.1007/s00266-018-1200-4

**Published:** 2018-08-17

**Authors:** Elena Gubanova, May Haddad Tabet, Yvonne Bergerova, Olena Moiseieva, Andrey Chemeris, Elena Sanches, Alisa Sharova, Luisa Rodriguez Pose, Romain Raymond, Inna Prygova, Ian Carlisle

**Affiliations:** 1 Vallex Med Clinic of Preventive Medicine, Moscow National University of Food Production, Starokaluzhskoe Shosse, 62, Moscow, Russian Federation 117630; 2 Dr Haddad Clinics, Beirut, Lebanon; 3 BcD Clinic, Prague, Czech Republic; 4 Clinic of Aesthetic Medicine, Ankor, Kyiv, Ukraine; 5 Department of Traditional Medicine, Central City Clinical Hospital, Almaty, Kazakhstan; 6 Medical Center of Aesthetic Correction “Eklan”, Moscow, Russian Federation; 7 Aesthetic Medicine “Chistie prudi”, Moscow, Russian Federation; 8 Ipsen Pharma, Boulogne-Billancourt, France; 9 Biostatistics, Ividata, Levallois-Perret, France; 10 Erase Aesthetic Services, Malvern, VIC Australia

**Keywords:** AbobotulinumtoxinA, Dysport^®^, Glabellar lines, Subject satisfaction, Physician satisfaction

## Abstract

**Background:**

Although the short- and long-term effectiveness of abobotulinumtoxinA (Dysport^®^/Azzalure^®^) for glabellar line (GL) treatment is well established, reporting of subject satisfaction over repeat treatment cycles is limited. The APPEAL study aimed to assess subject satisfaction with long-term GL treatment with abobotulinumtoxinA in a real-life setting.

**Methods:**

APPEAL was a noninterventional, prospective, longitudinal study in subjects administered ≥ 3 abobotulinumtoxinA injection cycles for moderate-to-severe GL, according to routine clinical practice. Subjects completed a satisfaction questionnaire at 3 weeks (± 7 days) after each cycle. Primary endpoint included subjects’ overall satisfaction with GL after three injection cycles. Secondary endpoints included satisfaction after Cycles 1 and 2 and factors associated with satisfaction after each cycle. Physician satisfaction was also assessed after Cycles 1 and 3.

**Results:**

Of 150 subjects enrolled, 135 completed the overall subject satisfaction questionnaire after Cycle 3. At 3 weeks after Cycle 3, 99.3% of subjects were ‘very satisfied’ (74.1%) or ‘satisfied’ (25.2%) with GL. Levels of subject satisfaction and associated factors after Cycles 1 and 2 were as large and significant as after Cycle 3 (83–100%, depending on question). Physicians’ satisfaction with GL appearance, facial expression, and overall satisfaction was almost complete after the first injection (≥ 97.4%) and unanimous after the third (100%).

**Conclusions:**

In the APPEAL study, overall satisfaction was high after three abobotulinumtoxinA injection cycles for GL based on both subjects’ (99.3%) and physicians’ (100.0%) assessments. High levels of subject satisfaction reported after Cycle 1 were maintained with repeated injections. No new safety signals were observed.

**Level of Evidence IV:**

This journal requires that authors assign a level of evidence to each article. For a full description of these Evidence-Based Medicine ratings, please refer to the Table of Contents or the online Instructions to Authors www.springer.com/00266.

*Trial registration* NCT02353897

**Electronic supplementary material:**

The online version of this article (10.1007/s00266-018-1200-4) contains supplementary material, which is available to authorized users.

## Introduction

Prolonged hyperactivity of facial muscles that results in the pleating of overlying skin can lead to permanent wrinkles, such as glabellar lines (GL) [1]. The presence of such hyperfunctional facial lines can have social and psychological implications as they can cause erroneous negative facial expressions to be conveyed, such as anger, anxiety, fear or sadness, and are associated with the external signs of aging [1, 2].

Both physicians and individuals considering aesthetic facial procedures recognize the vital role that facial expression has in self-perception, emotional well-being, and perception by others [1, 3]. Therefore, it is important to assess subject satisfaction and experience of treatment when evaluating the effectiveness of aesthetic procedures as this is a key feature of treatment success [4].

Intramuscular injections of botulinum neurotoxin type A (BoNT-A) inhibit acetylcholine release, causing temporary paralysis of the treated hyperfunctional muscles [3]. AbobotulinumtoxinA (Dysport^®^, Ipsen Biopharm Ltd., Wrexham, UK; Azzalure^®^, Galderma Ltd., Lausanne, Switzerland), a preparation of BoNT-A, has been approved for the treatment of moderate-to-severe GL across Europe and the USA since 2009. Although the short- and long-term safety and effectiveness of abobotulinumtoxinA for the treatment of GL is well established [5–9], a thorough investigation of subject satisfaction over repeat treatment cycles has not been performed [10].

The APPEAL study aimed to assess subject satisfaction with long-term GL treatment with abobotulinumtoxinA in a real-life setting. In real-life practice, physicians tailor treatment, taking into account the muscle structure (i.e., length and strength of the corrugator), the wrinkle severity and subject preference for a more natural or more static look [11]. Thus, the present study also aimed to record injection practices of participating physicians and their satisfaction with abobotulinumtoxinA treatment.

## Materials and Methods

### Objectives

The primary objective of the APPEAL study was to assess subject satisfaction with the appearance of their GL after three injection cycles of abobotulinumtoxinA.

Secondary objectives of this study were:

- Subject assessment of:
  - Individual treatment expectations;
  - Satisfaction after one and two injection cycles of abobotulinumtoxinA;
  - Factors associated with subject satisfaction including attractiveness, self-esteem, self-perceived age, and desire to receive another injection after each injection cycle.
- Physician assessment of:
  - GL severity at baseline and at injection visit 3 (at rest and maximum frown);
  - Satisfaction after one and three injection cycles of abobotulinumtoxinA at follow-up visits (if performed).
- To describe abobotulinumtoxinA injection practices.

### Study Design and Treatment

The APPEAL study (NCT02353897) was an international, noninterventional, prospective, longitudinal study conducted in 13 centers across six countries (Australia, Czech Republic, Kazakhstan, Lebanon, Russian Federation, and Ukraine) with marketing authorization for abobotulinumtoxinA approved for the treatment of GL.

This study was conducted in compliance with the Declaration of Helsinki, 2008. Prior to study initiation, approval was obtained from the independent ethics committee (IEC) or institutional review board (IRB) as applicable for each country involved. All subjects provided written informed consent to participate in this study prior to enrollment. Subjects did not receive any remuneration for their participation in this study. As this study was noninterventional, the decision to receive long-term (≥ 3 cycles) treatment with abobotulinumtoxinA for GL must have been made prior to and independently of the decision to participate in this study.

AbobotulinumtoxinA was administered in accordance with routine clinical practice, and the shortest treatment interval was in accordance with the local summary of product characteristics (SmPC). The maximum recommended treatment interval was < 6 months. Details of treatment administration were documented in an electronic case report form. This study was initiated in October 2014 and completed in December 2016.

**Fig. 1:**
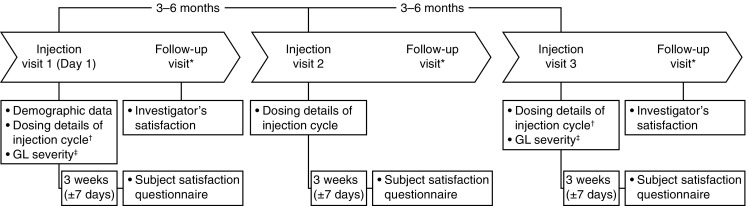
Study design and schedule of assessments. *Follow-up visits only if part of the investigator’s normal practice. ^†^Including muscles injected, total dose, total volume, and number of injection points. ^‡^At rest and at maximum frown

The study design and schedule of assessments are shown in Fig. 1. Subjects attended three injection visits, and if it was the physician’s normal practice, they attended a follow-up visit at 3 weeks (± 7 days) post-injection. Subjects were also required to complete a subject satisfaction questionnaire at 3 weeks (± 7 days) after each injection.

### Inclusion and Exclusion Criteria

Subjects were eligible to participate in the present study if they had provided written informed consent and were adults aged ≥ 18 or 21 years, depending on local legislation, with moderate-to-severe GL (assessed using the GL severity scale [GLSS] [9, 12]) and naïve to any type of aesthetic treatment or procedure for GL (invasive or noninvasive).

Subjects were excluded from entering the study if they were hypersensitive to abobotulinumtoxinA or its excipients, had participated in an interventional trial within 30 days before enrollment, had an infection at the proposed injection points or presented with myasthenia gravis, Eaton Lambert syndrome or amyotrophic lateral sclerosis, or were at risk of any precautions, warning or contraindications specified in the local SmPC. Women were excluded if they were pregnant, nursing, or planning a pregnancy during the study.

### Outcome Measures

Subjects were required to complete an electronic subject satisfaction questionnaire using a web interface at 3 weeks (± 7 days) after each injection cycle (Supplementary Table 1A–C). The purpose of this questionnaire was to assess the subjects’ overall treatment satisfaction (Cycle 3 only), expectations for treatment (Cycle 1 only), and satisfaction with treatment after each injection cycle.

The primary effectiveness endpoint was subject satisfaction with GL following the third injection cycle (Supplementary Table 1C). This was calculated in two ways:

- Overall satisfaction, based on Question 8 of the questionnaire after Cycle 3, analyzed as a dichotomized variable (satisfied versus not satisfied) as well as with the 5-point Likert scale.
- Subject’s individual satisfaction and factors related to satisfaction, based on Question 1 to Question 7 of the questionnaire.

Secondary effectiveness endpoints in relation to the subject assessment of treatment were:

- Subject’s individual expectations for treatment assessed after one injection cycle only (Supplementary Table 1A).
- Subject satisfaction after one and two injection cycles of abobotulinumtoxinA, assessed using the 5-point Likert scale (Supplementary Table 1A and B).
- Factors associated with subject satisfaction after each injection cycle (Supplementary Table 1A–C), including aesthetic outcome, self-perceived age, natural look, expectations met, self-feeling improvement, recommendation to family or friends, and desire to receive another injection.

Secondary effectiveness endpoints in relation to the physician assessment of treatment at injection and follow-up visits were:

- GL severity (at rest and at maximum frown) at baseline and injection visit 3 as per the investigator’s usual practice, using the GLSS (0, none; 1, mild; 2, moderate; 3, severe) [9, 12].
- Physician satisfaction with GL during follow-up visits (if performed) after one and three injections cycles of abobotulinumtoxinA. This was assessed using a 5-point Likert scale for satisfaction.

A description of injection practices was recorded at each injection visit, including muscle injected, total dose and volume injected per muscle, number of injection points, and interval between injections.

### Safety Reporting

As this was a noninterventional study, investigators were required to report all serious adverse events (AEs) and all related AEs (serious or non-serious) to Ipsen Pharmacovigilance department. AE data were included in the global drug safety database, but were not part of the clinical database.

### Statistical Analyses

The sample size was calculated based on the primary endpoint expressed as a proportion of satisfaction (i.e., very satisfied or satisfied): assuming 80% of subjects were very satisfied or satisfied after three injection cycles, 150 subjects would allow an estimation of this proportion with a precision of ± 6.4%. An interim analysis of baseline data was performed when at least one-third of the subjects had completed the first satisfaction questionnaire after injection Cycle 1. The cut-off date of the interim analysis was April 30, 2015, and data from 58 subjects were analyzed. These data were reanalyzed at the time of the final analyses.

Analyses were performed on the full study (FS) population (all subjects with a signed informed consent form and who received at least one injection). A supportive analysis of the primary effectiveness endpoint was based on the per protocol (PP) population (subjects with no major protocol violations/deviations). Descriptive summary statistics (n, mean, standard deviation, median, minimum and maximum) or frequency counts (subject numbers and percentages) were performed for the FS and PP populations for all subject satisfaction and questionnaire data as well as investigator satisfaction data and GL severity status. For satisfaction proportions, 95% confidence intervals (95% CI) were calculated using the Agresti–Coull method for approximate binomial CIs.

All statistical analyses were performed using Statistical Analysis System (SAS^®^) version 9.4 (SAS Institute Inc., Cary, NC, USA).

## Results

### Patient Disposition and Baseline Characteristics

In total, 150 subjects (13 males and 137 females) were recruited. All subjects received ≥ 1 injection of abobotulinumtoxinA and were thus included in the FS population. Of these 150 subjects, 137 (91.3%) completed the study (i.e., three complete abobotulinumtoxinA treatment cycles) and 13 subjects (8.7%) withdrew from the study due to consent withdrawal (*n* = 5), lack of satisfaction (*n* = 1), lost to follow-up (*n* = 1), or other reasons (*n* = 6).

Fifteen subjects (10.0%) were excluded from the PP population (*N* = 135, 90.0%) due to protocol deviations. These 15 subjects did not complete the subject satisfaction questionnaire for at least one visit and did not answer Question 8 of the subject satisfaction questionnaire after injection visit 3 (primary endpoint). Thus, the PP population was the same as the FS population with non-missing values.

Demographic and baseline characteristics are presented in Table 1. Overall, 91.3% of subjects enrolled were female and 38.7% of subjects were aged 41–50 years, with a similar distribution of subjects between the 31–40 years (21.3%) and 51–60 years (22.7%) age groups. Facial asymmetry was reported in ten subjects (6.7%) prior to treatment. All subjects were naïve for any type of aesthetic treatment or procedure (invasive and noninvasive) for GL.

**Table 1:** Demographic and baseline characteristics

	Full study population (*N* = 150), *n* (%)
*Sex*	
Male	13 (8.7%)
Female	137 (91.3%)
*Age (years)*	
18–30	13 (8.7%)
31–40	32 (21.3%)
41–50	58 (38.7%)
51–60	34 (22.7%)
> 60	13 (8.7%)
*Pre-treatment facial asymmetry*	
Yes	10 (6.7%)
No	140 (93.3%)

### Primary Endpoint: Overall Subject Satisfaction after Three Injection Cycles

**Fig. 2:**
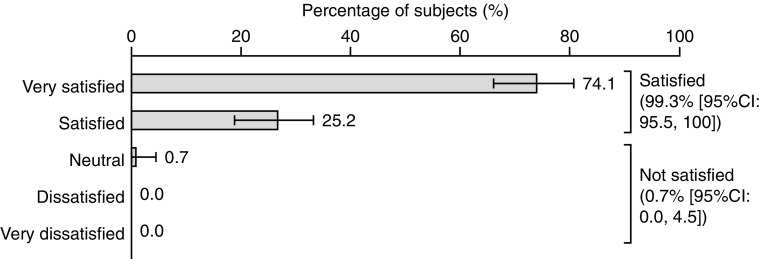
Assessment of overall subject satisfaction after three injection cycles (Likert scale). Values are presented as the percentage (95% confidence interval). Percentages are based on the number of subjects in the full study population with non-missing values (*n* = 135). Dichotomized modalities for treatment satisfaction defined as: satisfied = very satisfied + satisfied; not satisfied = neutral + dissatisfied + very dissatisfied

At 3 weeks (± 7 days) following a third injection cycle with abobotulinumtoxinA, 99.3% of subjects with available data (*n* = 135) responded that they were ‘very satisfied’ (74.1%, 95% CI 66.1; 80.8%) or ‘satisfied’ (25.2%, 95% CI 18.6; 33.2%) in response to the question ‘what is your overall satisfaction of the treatment?’ (Fig. 2). Only one subject (0.7%, 95% CI 0.0; 4.5) responded ‘neutral’ in response to this question.

This high proportion of subjects with overall satisfaction is reflected by responses to the individual factors relating to treatment satisfaction (Table 2). After three injection cycles, positive ratings were received for each factor in > 92% of subjects with particularly high ratings achieved for aesthetic outcome (99.3%), natural looks (100.0%), recommendation to friends and family (99.3%), and happy to receive the same treatment again (98.5%).

**Table 2:** Assessment of factors related to subject satisfaction after each injection cycle

	After one injection (*N* = 150)	After two injections (*N* = 150)	After three injections (*N* = 150)
Number evaluable patients	135	112	135
Happy to receive treatment again (yes)	131 (97.0)	112 (100.0)	133 (98.5)
Would recommend to family or friends (yes)	132 (97.8)	112 (100.0)	134 (99.3)
Self-feeling improvement (little, much or lot better)	125 (92.6)	108 (96.4)	128 (94.8)
Meets or exceeds expectations	128 (94.8)	109 (97.3)	132 (97.8)
Natural looks (yes)	133 (98.5)	111 (99.1)	135 (100.0)
Self-perceived age (looked much or a little younger)	112 (82.9)	99 (88.4)	124 (91.9)
Very satisfied or satisfied with aesthetic outcome in injected area	131 (97.0)	108 (96.4)	134 (99.3)

Data are presented as *n* (%)

Percentages are calculated based on the number of subjects with evaluable data. Full details are provided in Supplementary Table 3

After three injections, only one subject gave a neutral (neither satisfied nor dissatisfied) response to the aesthetic outcome in the injected area, and no subjects considered they looked older, that their expectations were not met, that the results of treatment did not look natural or that their feelings about themselves had worsened (Table 2; Supplementary Table 3).

### Subject Expectations for Treatment

Expectations for treatment were recorded in the satisfaction questionnaire through six questions (Questions 1, 2, 5, 8, 9 and 11; Supplementary Table 1A) addressed in the first subject questionnaire after one injection cycle with abobotulinumtoxinA. This questionnaire was completed by 135 subjects.

The primary reason for receiving treatment was a personal wish related to appearance or attractiveness in 124 (91.9%) subjects. Overall, 125 (92.6%) subjects were ‘very satisfied’ or ‘satisfied’ with injection comfort, 117 (86.7%) reported a rested appearance and 119 (88.1%) felt more attractive after one injection cycle (Supplementary Table 2).

In response to a multiple-choice question (Question 8; Supplementary Table 1A), subjects responded that injections brought them more harmony (45.2%, 61/135), self-esteem (41.5%, 56/135), and youth (40.0%, 54/135). Beauty (32.6%, 44/135) and symmetrical appearance (23.0%, 31/135) were also selected, though with a lower frequency (Supplementary Table 2).

Positive feedback from relatives was received by 104 (77.0%) subjects, while 27 (20.0%) received no feedback and four (3.0%) received both positive and negative feedback (Supplementary Table 2).

### Subject Satisfaction after One and Two Injection Cycles

The subject satisfaction questionnaire was completed by 135 subjects after one injection cycle and 112 subjects after two injection cycles. Subjects’ responses to factors related to satisfaction after one and two injection cycles are reported in Table 2 and Supplementary Table 3.

Overall, the level of subject satisfaction after the first and second abobotulinumtoxinA injection cycles (ranging from 83 to 100%, depending on question) was comparable with the high levels of satisfaction reported after the third injection (92–100%). Notably, for the question relating to self-perceived age (looking younger), the proportion of positive responses increased across injection cycles (Table 2; Supplementary Table 3).

Across the three cycles, only one subject (after one injection) was dissatisfied with treatment, and one subject (after one injection) considered they looked older. Few subjects felt that their expectations were not met or that the results of treatment did not look natural, and only two subjects (after one injection) reported that their feelings about themselves had worsened (Table 2; Supplementary Table 3).

### Physician Assessment of GL

**Fig. 3:**
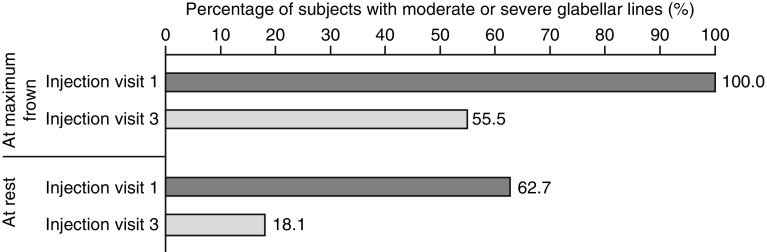
Physician assessment of glabellar line severity at maximum frown and at rest at injection visits 1 and 3. Values are presented as the percentage of subjects assessed as having moderate or severe glabellar lines. Percentages are based on the number of subjects in the full study population with non-missing values (*n* = 135). Further details are available in Supplementary Table 3

GL severity, at rest and at maximum frown, recorded at baseline (injection visit 1) and injection visit 3, showed improvements as per physician assessments (Fig. 3; Supplementary Table 4).

GL severity at rest was assessed as moderate to severe in 62.7% of subjects (94/150 subjects: moderate, 46.0%; severe, 16.7%) at baseline, compared with only 18.1% (25/138 subjects: moderate, 16.7%; severe, 1.4%) at injection visit 3. As a requirement of study enrollment, GL severity at maximum frown was rated as moderate to severe in all subjects at baseline (150/150 subjects: moderate, 54.0%; severe, 46.0%); this decreased to 55.1% (76/138 subjects: moderate, 47.8%; severe, 7.2%) at injection visit 3.

### Physician Satisfaction after One and Three Injection Cycles

**Fig. 4:**
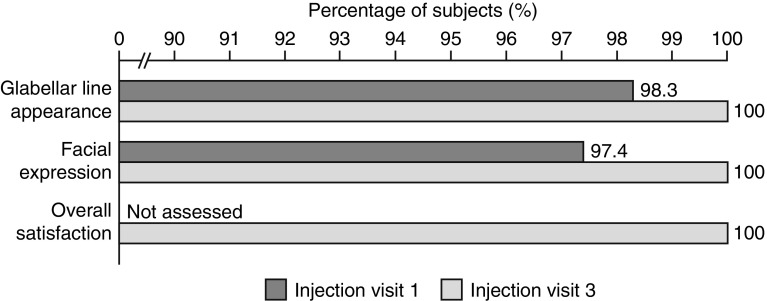
Physician satisfaction after one and three injection cycles. Percentage of physicians who were satisfied with each factor. Dichotomized 5-point Likert scale for treatment satisfaction: satisfied = very satisfied + satisfied; not satisfied = neutral + dissatisfied + very dissatisfied. Further details are available in Supplementary Table 4

Physicians reported they were ‘very satisfied’ or ‘satisfied’ with outcomes in more than 97% of their subjects regarding GL appearance (98.3%, *n* = 115: very satisfied, 68.4%; satisfied, 29.9%) and facial expression (97.4%; *n* = 114: very satisfied, 62.4%; satisfied, 35.0%) after the first injection cycle compared with before abobotulinumtoxinA injections (Fig. 4 and Supplementary Table 5).

After three injection cycles, physicians were ‘very satisfied’ or ‘satisfied’ with outcomes for all of their subjects (100%, *N* = 101) regarding GL appearance (very satisfied, 80.2%; satisfied, 19.8%), facial expression (very satisfied, 76.2%; satisfied, 23.8%), and overall satisfaction (assessed at follow-up visit 3 only: very satisfied 82.2%; satisfied 17.8%).

### Injection Practices

Details of the median injection doses and volumes and injection points are presented in Table 3. At each injection visit, the overall median [range] dose injected was 50 U [10–70 U, injection visits 1 and 2; 24–70 U, injection visit 3], corresponding to a median volume of 0.25 mL [0.05–0.40 mL, injection visits 1 and 2; 0.07–0.40 mL, injection visit 3] injected across the procerus and corrugator (left and right) muscles.

**Table 3:** AbobotulinumtoxinA injection practices in the APPEAL study

	All subjects (*N* = 150)								
	Procerus			Corrugator (left and right)			All muscles		
	Injection visit 1	Injection visit 2	Injection visit 3	Injection visit 1	Injection visit 2	Injection visit 3	Injection visit 1	Injection visit 2	Injection visit 3
*n*	138	130	127	149	139	137	150	140	137
*Total injected dose (U)*									
Mean (SD)	11.4 (2.9)	11.1 (2.6)	11.1 (2.6)	35.6 (7.7)	35.5 (7.0)	35.8 (7.2)	45.8 (7.5)	45.5 (6.8)	46.0 (6.7)
Median	10.0	10.0	10.0	40.0	40.0	40.0	50.0	50.0	50.0
Min, Max	5, 20	5, 20	5, 20	10, 60	20, 60	20, 60	10, 70	10, 70	24, 70
*Total injected volume (mL)*									
Mean (SD)	0.069 (0.039)	0.067 (0.040)	0.067 (0.040)	0.199 (0.061)	0.197 (0.059)	0.202 (0.061)	0.261 (0.084)	0.258 (0.082)	0.264 (0.082)
Median	0.05	0.05	0.05	0.20	0.20	0.20	0.25	0.25	0.25
Min, Max	0.01, 0.16	0.01, 0.16	0.01, 0.16	0.06, 0.32	0.08, 0.32	0.06, 0.32	0.05, 0.40	0.05, 0.40	0.07, 0.40
*Number of injection points*									
Mean (SD)	1.5 (0.7)	1.5 (0.8)	1.4 (0.8)	3.3 (1.1)	3.2 (1.1)	3.2 (1.2)	–	–	–
Median	1.0	1.0	1.0	4.0	4.0	4.0	–	–	–
Min, Max	1, 3	1, 3	1, 3	2, 6	2, 6	2, 6	–	–	–

*n* number of subjects with evaluable data, *SD* standard deviation

One point of injection was recorded in the procerus for 65–72% of subjects at each visit. These results were in accordance with the local SmPC, recommending a 10-U injection at one point. For corrugators, the local SmPC recommends a 10-U injection at two injection points for each corrugator muscle (left and right). Injectors aligned to this recommendation for 50–56% of subjects at each visit, while 40–46% of subjects received injections at only two injection points in total across the corrugator muscles. Median total injected units in both the procerus and corrugator muscles were in line with the local SmPC.

According to the product label [13], 11 subjects (17 events) received abobotulinumtoxinA injections over the recommended maximum dose of 50 U, ranging from 55 to 70 U.

The median (median in days [range]) time between injection cycles was 5.0 months (152 [85–267] days) between injection Cycles 1 and 2 (*N* = 140), and 5.3 months (161 [104–211] days) between injection Cycles 2 and 3 (*N* = 137).

### Safety

In this noninterventional study, abobotulinumtoxinA was administered and managed within routine medical care. In line with regulations related to noninterventional studies, investigators reported all serious AEs (related or not) and all non-serious AEs considered to be related to abobotulinumtoxinA.

In total, 47 AEs were reported in 32 subjects during the course of the study. Of the 47 AEs, 45 were non-serious events classed as ‘injury, poisoning and procedural complications,’ including product preparation (dilution) errors (20 events in 20 subjects, none of these 20 subjects reported AEs following the injection), wrong technique in product usage process (six events in six subjects; all received a total dose of 40 U across seven injection points), overdose (17 events in 11 subjects, three men and eight women; total injected dose of 55–70 U) and exposure during pregnancy (two events in two subjects; both subjects delivered a baby without complications).

The remaining two events (in two subjects) were non-serious AEs classed as ‘general disorders and administration site conditions,’ one event of drug ineffectiveness, and one case of injection-site pain reported as an AE (*n* = 1). The AE of injection-site pain was the only AE considered related to treatment and was resolved without sequelae. This subject was reinjected in the next injection cycle with abobotulinumtoxinA with no other AEs reported.

No deaths, serious AEs, or other significant AEs were reported during this study.

## Discussion

The results of the APPEAL study confirm that abobotulinumtoxinA was perceived as effective over three injection cycles in subjects treated for moderate-to-severe GL in real-life clinical practice. Although previous studies support positive outcomes for subject satisfaction with abobotulinumtoxinA for GL treatment [10], APPEAL is the first international, prospective study to observe these subject-perceived benefits following long-term abobotulinumtoxinA treatment. Importantly, these data were collected from investigators’ real-life clinical practice and so demonstrate long-term satisfaction with abobotulinumtoxinA from an alternative perspective compared with data collected from highly regimented RCTs. In this study, a high level of satisfaction (99.3%) was reported for the primary effectiveness endpoint, overall satisfaction with abobotulinumtoxinA treatment after three injection cycles. This level of satisfaction was supported by the high levels of subject satisfaction in response to questions about individual factors of treatment success (92–100%), and by the absolute (100%) overall satisfaction reported by physicians.

In the present study, 83–99% of subjects provided positive responses to questions regarding factors associated with treatment satisfaction after a single injection cycle of abobotulinumtoxinA. These high levels of subject satisfaction were maintained at Cycles 2 and 3.

These results were consistent with the overall satisfaction levels reported by Rzany et al. [5, 14] in a retrospective study of up to five injection cycles of abobotulinumtoxinA (*N* = 945). Rzany et al. reported consistently high levels of treatment satisfaction across injection cycles for both subjects (96–99%) and physicians (88–94%), rated on a 3-point scale (satisfactory, not satisfactory or unknown) [5, 10, 14]. This maintained level of subject satisfaction across cycles is also concordant with results reported by Ascher et al. [15] from a multicenter study of two injection cycles conducted in France, in which 78% of subjects either ‘completely satisfied’ or ‘satisfied’ with treatment at 1 month following initial injection and 85.4% after a second injection cycle of abobotulinumtoxinA (*N* = 50). Molina et al. [16] also reported high levels of overall subject satisfaction at 3 weeks following abobotulinumtoxinA treatment for GL in a single-cycle, noninterventional study. In the present study, and similarly to Molina et al., we report high levels of satisfaction with outcomes such as comfort of treatment (93%), appearing rested (87%), aesthetic outcome (96–99%), meeting or exceeding expectations (95–98%), natural look (99–100%), looking younger (83–92%) and self-feeling improvement (93–96%) [10, 16]. There was a tendency toward higher levels of subject satisfaction in the present study, which may be due to additional improvements observed with repeat treatment with abobotulinumtoxinA.

The dose administered to each subject was at the discretion of the investigator and in line with normal clinical practice. Median dose of abobotulinumtoxinA injected across all muscles was 50.0 U at each injection cycle, as recommended in the product label [13]. As discussed by De Almeida et al., the achievement of more effective and natural-looking results with botulinum toxin treatment for GL requires an understanding of the interpersonal differences in muscle contraction patterns and individualized treatment [11]. In this study, although median doses injected were in line with the product label, the noninterventional design allowed physicians the flexibility to tailor injection practices according to their clinical judgment in order to improve subject satisfaction with the result. However, satisfaction with abobotulinumtoxinA for GL treatment in the APPEAL study was observed across a very high proportion of subjects in a large study population, which suggests satisfaction is achieved regardless of independent investigator’s use of the product.

The noninterventional design of the APPEAL study means that the high levels of satisfaction observed here reflect real-life treatment and as such is a strength of this study. This type of study design can also be considered a limitation due to the less stringent criteria for enrollment compared with randomized controlled studies, which may limit the extent to which results can inform the treatment of specific participant groups. The lack of a placebo arm as a control group could also be considered a limitation of this study design. However, these limitations may be addressed by previous double-blind randomized controlled and open-label studies [14]. All participants included in the APPEAL study were naïve to aesthetic treatment for GL, which could have impacted the results to an extent, although the participation criteria are necessary to provide a sufficiently homogenous population. Thirteen patients withdrew from the study, only one of which was due to lack of satisfaction, and so the withdrawal of these patients is unlikely to have skewed the data. Subjects were not remunerated for their participation in this study and the decision to inject abobotulinumtoxinA was made independently from their decision to enroll, helping to minimize bias in this study.

The most common AEs observed were errors in product dilution, injection technique and overdose. Double-blinded, randomized trials of abobotulinumtoxinA at different doses could be useful for informing future practice in aesthetic medicine.

## Conclusions

In the APPEAL study, overall satisfaction levels were high (99.3%) following three injection cycles with abobotulinumtoxinA for moderate-to-severe GL when assessed by subjects and complete (100.0%) by physician assessment. Subjects reported high levels of treatment satisfaction after one injection with abobotulinumtoxinA, and this satisfaction was maintained after repeated injections. Doses of abobotulinumtoxinA administered were consistent across treatment cycles and physicians’ injection practice aligned to the SmPC. AEs were in line with the known BoNT-A profile, and no new safety signals were observed.

## Electronic supplementary material

Below is the link to the electronic supplementary material.

Supplementary material 1 (DOCX 32 kb)
