# Supplementary material for: Assessment of Subject and Physician Satisfaction after Long-Term Treatment of Glabellar Lines with AbobotulinumtoxinA (Dysport®/Azzalure®): Primary Results of the APPEAL Noninterventional Study
Source: Aesthetic Plast Surg. 2018 Aug 17;42(6):1672–80. doi: 10.1007/s00266-018-1200-4 (PMC6280832; doi:10.1007/s00266-018-1200-4)
Supplement: Supplementary file 1 — Supplementary material 1 (DOCX 32 kb) [file 266_2018_1200_MOESM1_ESM.docx]

**Assessment of subject and physician satisfaction after long-term treatment of glabellar lines with abobotulinumtoxinA (Dysport^®^/Azzalure^®^): Primary results of the APPEAL non-interventional study**

**Supplementary material**

**Table 1.** **Patient satisfaction questionnaire completed after each injection cycle.**

| 1. **Questions following the first injection cycle** | |
| --- | --- |
| 1. What is the main reason you decided to receive a corrective injection? | o Personal wish for my appearance or attractiveness  o Job requirement  o Spouse / friends / family pressure  o Other: _______________________________ |
| 1. How satisfied were you with the comfort of the injection? | o Very satisfied  o Satisfied  o Neutral (neither satisfied nor dissatisfied)  o Dissatisfied  o Very dissatisfied |
| 1. How satisfied have you been with the aesthetic outcome in the injected area, within the 3 weeks after injection? | o Very satisfied  o Satisfied  o Neutral (neither satisfied nor dissatisfied)  o Dissatisfied  o Very dissatisfied |
| 1. With which statement do you now agree? | o I look much younger than my age  o I look a little younger than my age  o I look my current age  o I look a little older than my age  o I look much older than my age |
| 1. Would you say that you appear rested as if you were coming back from vacation? | o Yes  o No |
| 1. Do you think that the result of the treatment looks natural? | o Yes  o No |
| 1. Would you say that the results of the injection: | o Surpasses my expectations  o Meets my expectations  o Does not meet my expectations  o I did not have any expectations |
| 1. Do you think that the injection brings you more:   *(tick all boxes that apply)* | □ Beauty  □ Harmony  □ Symmetrical appearance  □ Youth  □ Self-esteem /confidence  □ None of the above |
| 1. Would you say that you feel more attractive? | o Yes  o No |
| 1. How do you feel about yourself since the injection has been performed? | o Much better  o A lot better  o A little better  o The same  o Worse |
| 1. Did you get any feedback about your look from your family, friends, colleagues? | o Positive feedback  o Negative feedback  o Both positive and negative feedbacks  o No feedback |
| 1. Would you recommend this treatment to your family or friends? | o Yes  o No |
| 1. Would you be happy to receive the same treatment again? | o Yes  o No |
| 1. **Questions following the second injection cycle** | |
| 1. How satisfied have you been with the aesthetic outcome in the injected area within the 3 weeks after injection? | o Very satisfied  o Satisfied  o Neutral (neither satisfied nor dissatisfied)  o Dissatisfied  o Very dissatisfied |
| 1. With which statement do you now agree? | o I look much younger than my age  o I look a little younger than my age  o I look my current age  o I look a little older than my age  o I look much older than my age |
| 1. Do you think that the result of the treatment looks natural? | o Yes  o No |
| 1. Would you say that the results of the injection: | o Surpasses my expectations  o Meets my expectations  o Does not meet my expectations  o I did not have any expectations |
| 1. How do you feel about yourself since the injection has been performed? | o Much better  o A lot better  o A little better  o The same  o Worse |
| 1. Would you recommend this treatment to your family or friends? | o Yes  o No |
| 1. Would you be happy to receive the same treatment again? | o Yes  o No |
| 1. **Questions following the third injection cycle** | |
| 1. How satisfied have you been with the aesthetic outcome in the injected area within the 3 weeks after injection? | o Very satisfied  o Satisfied  o Neutral (neither satisfied nor dissatisfied)  o Dissatisfied  o Very dissatisfied |
| 1. With which statement do you now agree? | o I look much younger than my age  o I look a little younger than my age  o I look my current age  o I look a little older than my age  o I look much older than my age |
| 1. Do you think that the result of the treatment looks natural? | o Yes  o No |
| 1. Would you say that the results of the injections: | o Surpasses my expectations  o Meets my expectations  o Does not meet my expectations  o I did not have any expectations |
| 1. How do you feel about yourself since the injection has been performed? | o Much better  o A lot better  o A little better  o The same  o Worse |
| 1. Would you recommend this treatment to your family or friends? | o Yes  o No |
| 1. Would you be happy to receive the same treatment again? | o Yes  o No |
| 1. What is your overall satisfaction of the treatment? | o Very satisfied  o Satisfied  o Neutral (neither satisfied nor dissatisfied)  o Dissatisfied  o Very dissatisfied |

**Supplementary Table 2. Subject responses to questionnaire: expectations for treatment.**

| **Question** | | **Responses,**  **n (%) [95% CI]** |
| --- | --- | --- |
| What is the main reason you decided to receive a corrective injection? | Personal wish for my appearance or attractiveness | 124 (91.9)  [85.9, 95.5] |
|  | Job requirement | 2 (1.5)  [0.1, 5.6] |
|  | Spouse/friends/family pressure | 8 (5.9)  [2.9, 11.4] |
|  | Other | 1 (0.7)  [0.0, 4.5] |
| How satisfied were you with the comfort of the injection? | Very satisfied | 93 (68.9)  [60.6, 76.1] |
|  | Satisfied | 32 (23.7)  [17.3, 31.6] |
|  | Neutral (neither satisfied nor dissatisfied) | 10 (7.4)  [3.9, 31.6] |
|  | Dissatisfied | 0  [0.0, 3.3] |
|  | Very dissatisfied | 0  [0.0, 3.3] |
| Would you say that you appear rested as if you were coming back from vacation? | Yes | 117 (86.7)  [79.8, 91.5] |
|  | No | 18 (13.3)  [8.5, 20.2] |
| Do you think that the injection brings you more: | Beauty | 44 (32.6)  [25.2, 40.9] |
|  | Harmony | 61 (45.2)  [37.0, 53.6] |
|  | Symmetrical appearance | 31 (23.0)  [16.6, 30.8] |
|  | Youth | 54 (40.0)  [32.1, 48.4] |
|  | Self-esteem /confidence | 56 (41.5)  [33.5, 49.9] |
| Would you say that you feel more attractive? | Yes | 119 (88.1)  [81.5, 92.7] |
|  | No | 16 (11.9)  [7.3, 18.5] |
| Did you get any feedback about your look from your family, friends, colleagues? | Positive feedback | 104 (77.0)  [69.2, 83.4] |
|  | Negative feedback | 0  [0.0, 3.3] |
|  | Both positive and negative feedback | 4 (3.0)  [0.9, 7.6] |
|  | No feedback | 27 (20.0)  [14.1, 27.6] |

CI, confidence interval.

**Supplementary Table 3. Subject responses to questionnaire: factors related to subject satisfaction.**

| **Question** | | **Responses,**  **n (%) [95% CI]** | | |
| --- | --- | --- | --- | --- |
|  |  | **After one injection** | **After two injections** | **After three injections** |
| How satisfied have you been with the aesthetic outcome in the injected area within the 3 weeks after injection? | Very satisfied | 86 (63.7)  [55.3, 71.3] | 73 (65.2)  [56.0, 73.4] | 100 (74.1)  [66.1, 80.8] |
|  | Satisfied | 45 (33.3)  [25.9, 41.7] | 35 (31.3)  [23.4, 40.4] | 34 (25.2)  [18.6, 33.2] |
|  | Neutral (neither satisfied nor dissatisfied) | 3 (2.2)  [0.5, 6.6] | 4 (3.6)  [1.1, 9.1] | 1 (0.7)  [0.0, 4.5] |
|  | Dissatisfied | 1 (0.7)  [0.0, 4.5] | 0  [0.0, 4.0] | 0  [0.0, 3.3] |
|  | Very dissatisfied | 0  [0.0, 3.3] | 0  [0.0, 4.0] | 0  [0.0, 3.3] |
| With which statement do you now agree? | I look much younger than my age | 40 (29.6)  [22.6, 37.8] | 28 (25.0)  [17.9, 33.8] | 38 (28.1)  [21.2, 36.3] |
|  | I look a little younger than my age | 72 (53.3)  [44.9, 61.5] | 71(63.4%)  [54.2, 71.7] | 86 (63.7)  [55.3, 71.3] |
|  | I look my current age | 22 (16.3)  [10.9, 23.5] | 13 (11.6)  [6.8, 19.0] | 11 (8.1)  [4.5, 14.1] |
|  | I look a little older than my age | 1 (0.7)  [0.0, 4.5] | 0  [0.0, 4.0] | 0  [0.0, 3.3] |
|  | I look much older than my age | 0  [0.0, 3.3] | 0  [0.0, 4.0] | 0  [0.0, 3.3] |
| Do you think that the result of the treatment looks natural? | Yes | 133 (98.5)  [94.4, 99.9] | 111 (99.1)  [94.6, 100.0] | 135 (100.0)  [96.7, 100.0] |
|  | No | 2 (1.5)  [0.1, 5.6] | 1 (0.9)  [0.0, 5.4] | 0  [0.0, 3.3] |
| Would you say that the results of the injection: | Surpasses my expectations | 33 (24.4)  [17.9, 32.4] | 27 (24.1) [17.1, 32.8] | 40 (29.6)  [22.6, 37.8] |
|  | Meets my expectations | 95 (70.4)  [62.2, 77.4] | 82 (73.2)  [64.3, 80.6] | 92 (68.1)  [59.9, 75.4] |
|  | Does not meet my expectations | 4 (3.0)  [0.9, 7.6] | 3 (2.7)  [0.6, 7.9] | 0  [0.0, 3.3] |
|  | I did not have any expectations | 3 (2.2)  [0.5, 6.6] | 0  [0.0, 4.0] | 3 (2.2)  [0.5, 6.6] |
| How do you feel about yourself since the injection has been performed? | Much better | 39 (28.9)  [21.9, 37.1] | 31 (27.7)  [20.2, 36.6] | 53 (39.3)  [31.4, 47.7] |
|  | A lot better | 61 (45.2)  [37.0, 53.6] | 54 (48.2)  [39.2, 57.4] | 60 (44.4)  [36.3, 52.9] |
|  | A little better | 25 (18.5)  [12.8, 26.0] | 23 (20.5)  [14.0, 29.0] | 15 (11.1)  [6.7, 17.6] |
|  | The same | 8 (5.9)  [2.9, 11.4] | 4 (3.6)  [1.1, 9.1] | 7 (5.2)  [2.3, 10.5] |
|  | Worse | 2 (1.5)  [0.1, 5.6] | 0  [0.0, 4.0] | 0  [0.0, 3.3] |
| Would you recommend this treatment to your family or friends? | Yes | 132 (97.8)  [93.4, 99.5] | 112 (100.0)  [96.0, 100.0] | 134 (99.3)  [95.5, 100.0] |
|  | No | 3 (2.2)  [0.5, 6.6] | 0  [0.0, 4.0] | 1 (0.7)  [0.0, 4.5] |
| Would you be happy to receive the same treatment again? | Yes | 131 (97.0)  [92.4, 99.1] | 112 (100.0)  [96.0, 100.0] | 133 (98.5)  [94.4, 99.9] |
|  | No | 4 (3.0)  [0.9, 7.6] | 0  [0.0, 4.0] | 2 (1.5)  [0.1, 5.6] |

CI, confidence interval.

**Supplementary Table 4. Physician assessment of glabellar line severity before the first and third injection.**

| **Assessment** | **Moderate + severe glabellar lines, n (%)** | **Moderate or severe glabellar lines (%)** | **n (%),**  **[95% CI]** |
| --- | --- | --- | --- |
| **At maximum frown** | | | |
| Injection visit 1 (n=150) | 150 (100.0) | Moderate | 81 (54.0)  [46.0, 61.8] |
|  |  | Severe | 69 (46.0)  [38.2, 54.0] |
| Injection visit 3  (n=138) | 76 (55.1) | Moderate | 66 (47.8)  [39.7, 56.1] |
|  |  | Severe | 10 (7.2)  [3.8, 13.0] |
| **At rest** | | | |
| Injection visit 1  (n=150) | 94 (62.7) | Moderate | 69 (46.0)  [38.2, 54.0] |
|  |  | Severe | 25 (16.7)  [11.5, 23.5] |
| Injection visit 3  (n=138) | 25 (18.1) | Moderate | 23 (16.7)  [11.3, 23.8] |
|  |  | Severe | 2 (1.4)  [0.1, 5.5] |

CI, confidence interval.

**Supplementary Table 5. Physician satisfaction after one and three injection cycles.**

| **Assessment** | **Proportion of subjects with moderate or severe glabellar lines (%)** | | |
| --- | --- | --- | --- |
|  | **Satisfied + very satisfied, n (%)** | **Satisfied or very satisfied** | **n (%),**  **[95% CI]** |
| **Glabellar line appearance** | | | |
| Injection visit 1 (n=117) | 115 (98.3) | Satisfied | 35 (29.9)  [22.3, 38.8] |
|  |  | Very satisfied | 80 (68.4)  [59.5, 76.1] |
| Injection visit 3  (n=101) | 101 (100.0) | Satisfied | 20 (19.8)  [13.1, 28.7] |
|  |  | Very satisfied | 81 (80.2)  [81.3, 86.9] |
| **Facial expression** | | | |
| Injection visit 1  (n=117) | 114 (97.4) | Satisfied | 41 (35.0)  [27.0, 44.1] |
|  |  | Very satisfied | 73 (62.4)  [53.3, 70.7] |
| Injection visit 3  (n=101) | 101 (100.0) | Satisfied | 24 (23.8)  [16.5, 33.0] |
|  |  | Very satisfied | 77 (76.2)  [67.0, 83.5] |
| **Overall satisfaction** | | | |
| Injection visit 3  (n=101) | 101 (100.0) | Satisfied | 18 (17.8)  [11.5, 26.5] |
|  |  | Very satisfied | 83 (82.2)  [73.5, 88.5] |

CI, confidence interval.
